# Splenectomy for Visceral Leishmaniasis Out of an Endemic Region: A Case Report and Literature Review

**DOI:** 10.3390/medicina58020184

**Published:** 2022-01-26

**Authors:** Nebojsa Lekic, Boris Tadic, Vladimir Djordjevic, Dragan Basaric, Marjan Micev, Dragica Vucelic, Milica Mitrovic, Nikola Grubor

**Affiliations:** 1 Department for HBP Surgery, University Clinical Centre of Serbia, Clinic for Digestive Surgery, Koste Todorovica Street, No. 6, 11000 Belgrade, Serbia; nesalekic67@gmail.com (N.L.); vladimir.djordjevic@kcs.ac.rs (V.D.); dr.gale@mts.rs (D.B.); n.grubor@yahoo.com (N.G.); 2 Department for Surgery with Anesthesiology, Faculty of Medicine, University of Belgrade, Subotica No. 8, 11000 Belgrade, Serbia; 3 Department for Pathology, University Clinical Centre of Serbia, Clinic for Digestive Surgery, Koste Todorovica Street, No. 6, 11000 Belgrade, Serbia; micevm@gmail.com; 4 Department of Transfusion Medicine, Faculty of Medicine, University of Belgrade, Koste Todorovica Street, No. 6, 11000 Belgrade, Serbia; vucelicd@gmail.com; 5 Center for Radiology and Magnetic Resonance Imaging, University Clinical Centre of Serbia, Pasterova No. 2, 11000 Belgrade, Serbia; dr_milica@yahoo.com

**Keywords:** visceral leishmaniasis, impaired hemostasis, thromboelastometry, splenectomy

## Abstract

Visceral leishmaniasis (also known as kala-azar) is characterized by fever, weight loss, swelling of the spleen and liver, and pancytopenia. If it is not treated, the fatality rate in developing countries can be as high as 100% within 2 years. In a high risk situation for perioperative bleeding due to severe thrombocytopenia/coagulopathy, we present a rare challenge for urgent splenectomy in a patient with previously undiagnosed visceral leishmaniasis. A histologic examination of the spleen revealed a visceral leishmaniasis, and the patient was successfully treated with amphotericin B.

## 1. Introduction

Visceral leishmaniasis (VL) is a severe and potentially fatal systemic vector-borne zoonotic disease caused particularly by the protozoan species *Leishmania infantum* (syn. *Leishmania chagasi*) and *Leishmania donovani* [1]. It has an endemic character in tropical and subtropical countries. According to the latest data from World Health Organization, more than 90% of new cases in 2019 occurred in 10 countries, namely, Brazil, Ethiopia, Eritrea, India, Iraq, Kenya, Nepal, Somalia, and Sudan [2]. In Europe, VL occurs in rural regions of the Mediterranean and the Balkans, with 75% of the overall cases diagnosed in Italy, Spain, Albania, and Georgia [3]. In the middle of the last century, leishmaniasis was an endemic disease in Serbia. It has been considered to have been eradicated for more than 40 years, as the last case of VL was reported in 1968 [4].

**Figure 1:**
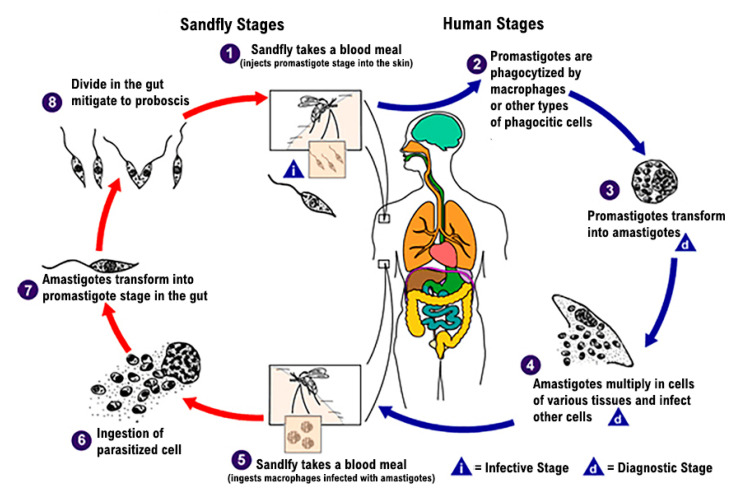
Lifecycle of *Leishmania* spp. [6].

The *Leishmania* parasite exists in the following two distinct forms: a promastigote form found in the vector, and an amastigote form, which develops intracellularly in the susceptible mammalian host. The infection is transmitted through the bites of infected female hematophagous sandflies (*Diptera: Psychodidae*, *Phlebotominae*). Insects feed on blood that contains macrophages infected with amastigotes to produce eggs. In the sandfly’s midgut, over 6–9 days, the parasites differentiate into promastigotes and multiply [5]. Thereafter, during the next blood meals, the sandflies inject the infective promastigotes into the host and they are phagocytized by macrophages (Figure 1) [6].

Patients with VL present symptoms of systemic infection. As the disease advances, hepatosplenomegaly may develop, causing bloating or discomfort in the upper portion of the abdomen. Moreover, splenomegaly may be accompanied by hypersplenism, anemia, thrombocytopenia, and occasional bleeding. Visceral leishmaniasis is a deadly disease, so if not treated effectively or misdiagnosed, it can be fatal [7].

In this report, we present a patient with fever, weight loss, splenomegaly and hypersplenism of uncertain origin, with the main intention to raise the awareness of possibility of this potentially fatal infective disease, which may not be endemic to the clinician’s geographical area.

## 2. Case Report

**Figure 2:**
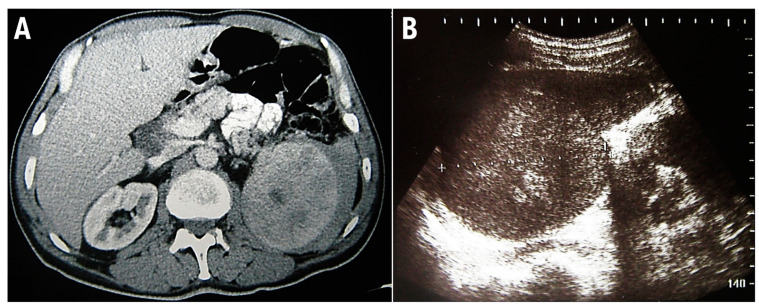
Multidetector computed tomography reveals enlarged, inhomogeneous spleen with intraparenchymal confluent zones of lower density, and a small amount of free fluid is present along the lateral contour of the spleen (**A**). Abdominal ultrasonographic examination shows enlarged spleen with diffusely inhomogeneous parenchyma (**B**).

This study conforms to the CARE guidelines [8]. A 48-year-old man was admitted for a prompt surgical consultation at the Clinical Center of Serbia, Belgrade, a clinic for abdominal surgery. Over the previous 3 months, the patient had experienced episodes of abdominal pain, malaise, fever, and repeated nose bleeding accompanied by weight loss of around 20 kg. The patient provided anamnestic information that during the last year he worked on a cruise ship on the Mediterranean route, including North African countries. On admission, no signs of hemorrhagic diathesis were observed. Abdomen ultrasonography and computed tomographic scanning revealed significant splenomegaly (Figure 2).

Based on the results of a full blood count, the patient was identified preoperatively as having anemia and severe thrombocytopenia (Table 1). In the patient’s samples, we performed two methods of hemostasis, including standard hemostasis tests and thromboelastometry (TEM^®^ International GmbH, Munich, Germany). We conducted extrinsically (EXTEM test: 20 µL CaCl_2_ 0.2 mmol/L, 20 µL rabbit brain tissue factor, 300 µL citrated whole blood) and intrinsically (INTEM test: 20 µL CaCl_2_ 0.2 mmol/L, 20 µL ellagic acid, 300 µL citrated whole blood) activated tests. For the differential diagnosis of fibrinogen/platelets’ contribution to clot firmness, a FIBTEM test (activation as in EXTEM with the addition of substance cytochalasin D, a strong inhibitor of the platelet cytoskeleton) was also analyzed.

The patient was found to be hypocoagulable, as suggested by standard hemostasis tests (prolonged activated partial thromboplastin time (APTT)/international normalized ratio (INR), decreased levels of coagulation factors (F) II, V, VII, X, XI, XIII) (Table 1).

The results of the various thromboelastometric parameters in different tests are shown in Table 2. In extrinsic and intrinsic activated tests, the CFT (clot formation time, defined as early fibrin polymerization) values were increased while MCF (maximum clot firmness, reflecting the absolute strength and stability of the fibrin clot) values were decreased, indicating a hypocoagulable state with the major contribution of a low platelet count to reduced clot firmness. In both tests, fibrinolityc activity was normal (not presented in Table 2).

**Figure 3:**
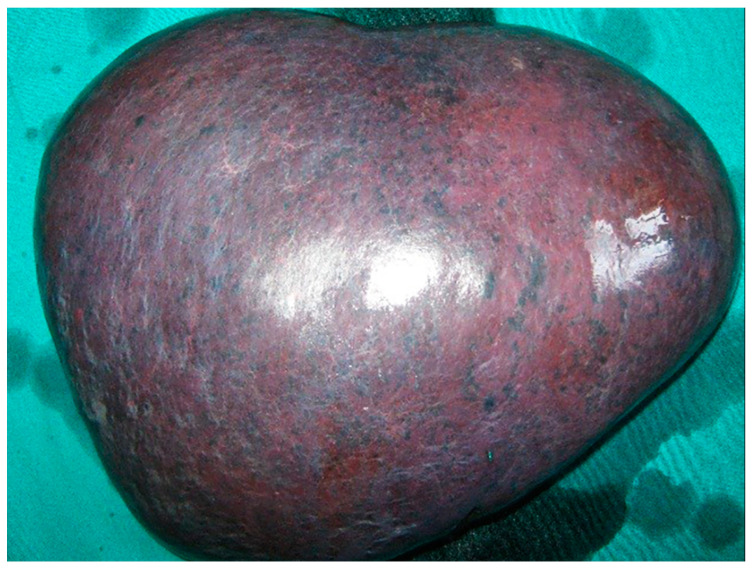
Macroscopic specimen of the removed spleen, 23 × 18 cm in size.

**Figure 4:**
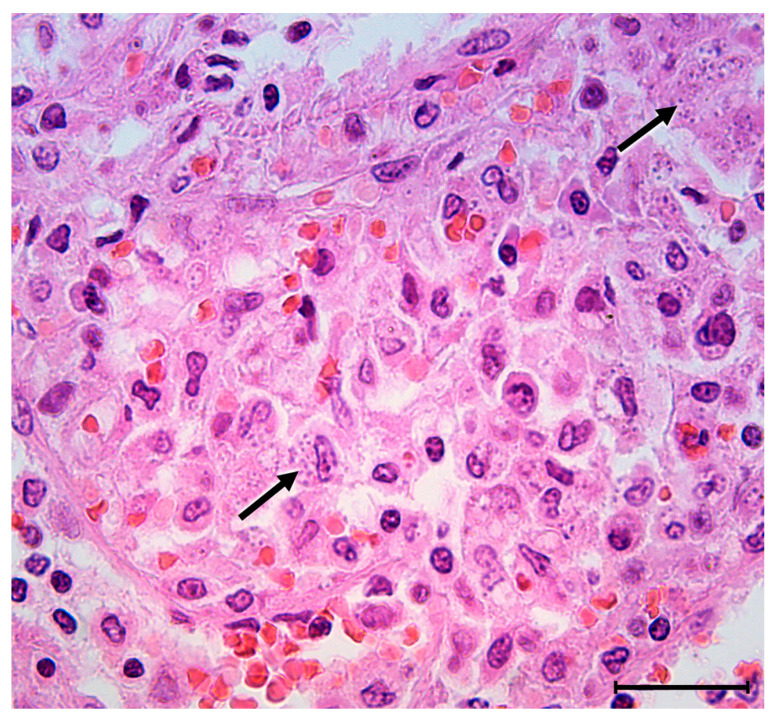
Microscopic examination on higher magnification reveals scattered groups of macrophages (upper right and lower left—see arrows) filled with coarse cytoplasmatic granules consistent with amastigotes of *Leishmania* protozoan (H&E staining, original magnification 63×, scale bar = 50 µm).

Despite severe thrombocytopenia/deteriorated hemostasis, the patient underwent an urgent splenectomy due to rapid clinical deterioration. Surgery was performed via a subcostal incision. The spleen weighed 3180 g, and was 23 × 18 cm in size (Figure 3). Subsequent microscopic examination revealed scattered groups of cells filled with coarse cytoplasmatic granules consistent with the amastigotes of the *Leishmania* protozoan (Figure 4).

Decision making for the hemostatic preoperative treatment was guided by these thromboelastometric results. In order to minimize blood loss, we constructed a preoperative prophylactic treatment strategy including the administration of vitamin K (in a total dose of 20 mg), desmopressin (1-deamino-8-D-arginine vasopressin (DDAVP)), and tranexamic acid.

DDAVP was used at a standard dose of 0.3 µg/kg in a 40 min saline infusion an hour before operation and once daily until the end of the second post-operative day. DDAVP administration was complemented with tranexamic acid administration in a dose of 1 g (diluted in 100 mL of isotonic saline and infused for 30 min) before surgery, and continued with the same dose in 8 h intervals until the end of the first post-operative day. Intraoperatively, the patient received one unit of leucodepleted red blood cells and pooled platelet concentrate (PC) (administered immediately after operative blood vessel ligation). No side effects as a result of hemostatic drug administration were recorded during or after surgery.

After the operation, the patient was admitted to the intensive care unit, where all vital parameters were closely monitored for 48 h. The postoperative hemoglobin concentration was 87 g/L, and platelet count was 27 × 10^9^/L. No bleeding complications were observed after surgery. The patient required no allogeneic blood products during the postoperative period. Low molecular weight heparin in prophylactic doses was introduced when the platelet count reached a value of 50 × 10^9^/L. The patient had an uneventful postoperative course and left the hospital on the 14th postoperative day. On discharge, their hemoglobin concentration and platelet count were 108 g/L and 82 × 10^9^/L, respectively. Following discharge, our patient was transferred to the clinic for infective diseases where he underwent conservative treatment, including amphotericin B. One year after splenectomy, the patient was symptom free and doing well.

## 3. Discussion

Visceral leishmaniasis, also called kala-azar, is an under-researched parasitic disease and is endemic to arid parts of the world, tropical and subtropical developing countries, and malnourished populations. Mass migration or travel to endemic areas may accelerate the development and spread of the disease [8]. According to the medical literature, VL is reported predominantly among males [9,10]. It is a severe and potentially fatal zoonotic disease with up to 90,000 new cases reported worldwide annually and a mortality rate of 95% in untreated patients [2]. However, in the endemic regions, a diagnosis or suspicion of VL based on clinical presentation is very challenging to arrive at. It mainly affects multiple organs of the reticuloendothelial system (RES), the lymph nodes, spleen, liver, and bone marrow, making it a systemic infection very similar to other infections or disorders simulating VL.

Leishmaniasis is a typically vector-borne, obligatory intracellular protozoan that replicates only inside of host cells. It is most commonly transmitted via the bite of the tiny hematophagous female sandflies of the genus *Phlebotomus* and *Lutzomyia*. However, there is evidence of different non-vector bloodborne transmission pathways. Congenital *Leishmania* transmission, from a mother to her child, regardless of manifested mother symptomatology is occasionally reported [11,12,13,14,15]. An interesting case report from 1960 suggests that leishmaniasis occurred as a result of intercourse [16]. Although this mode of transmission has been proven in dogs, there is still no strong evidence for humans [17]. Additionally, people who underwent organ transplantation are at risk of microorganisms transmission. However, the number of leishmaniasis cases resulting from organ transplants is not large (fewer than 100 in the literature). They are mostly individuals who have undergone kidney, liver, heart, lung, pancreas, stem cell, and bone-marrow transplants [18]. Blood transfusions are performed for various indications, so in patients with a history of blood transfusion, free of the other risk factors, leishmaniasis is generally considered to be a complication of the transfusion process [19,20]. Infection is also occasionally reported as a consequence of laboratory parenteral transmissions and from experimental animal bites [21].

After an incubation period lasting between 2 and 6 months, VL patients present symptoms and signs of persistent and very progressive systemic infection that occurs over weeks or even months [22]. Chronic intermittent fever is typically associated with rigor and chills. Enlarged spleen, liver, and lymph nodes occur as a consequence of the parasitic invasion of the blood and RES. Anemia is generally common in parasitic infections. Compromising the ability to supply oxygen to the tissues, it may play a role in causing other symptoms typical of these patients including malaise, weakness, fatigue, and pallor. Bone marrow is the major tissue for the production of erythrocytes and thrombocytes in humans. It is also one of the major sites for *Leishmania* invasion, so dyserythropoiesis is not unexpected since *Leishmania* infection often causes changes in cells [23]. Hemophagocytosis (histological appearance of blood-eating macrophages), a pathologic condition that can lead to anemia, has been widely reported in human VL patients [24,25].

In addition to anemia, patients with VL may represent a wide range of hematological abnormalities. Thrombocytopenia is very common, so it is not unexpected that patients with VL tend to bleed. Although the frequency of this complication, according to some authors, is less than 10%, bleeding is certainly a significant risk factor for fatal outcome in these patients. [26,27]. In our patient, severe thrombocytopenia could be attributed to increased splenic sequestration secondary to hypersplenism. However, in normal subjects, the spleen contains approximately one-third of the total platelet mass, and in the patient with splenomegaly 60–90% of the total platelet mass may be sequestered in the spleen. Patients with marked splenomegaly may not respond with an expected platelet count increment after transfusion (8000–1000 per µL per unit transfused in an average adult) because of the increased sequestration of transfused platelets in the enlarged spleen [28]. From this perspective, hemostatic drug administration in the perioperative period could be recognized as a measure in reducing perioperative blood loss and transfusion requirements [29,30].

DDAVP is a synthetic analog of the antidiuretic hormone vasopressin that induces an increase in plasma levels of von Willebrand factor (vWF) and FVIII three- to fivefold above baseline. In this way, DDAVP promotes platelet adhesion to the sub-endothelium and platelet–platelet interactions, as well as the coagulation process. These effects of DDAVP are likely explained by a direct action on the endothelium, via the activation of endothelial vasopressin V2 receptor and by activating cyclic adenosine monophosphate (cAMP)-mediated signaling [31]. Additionally, the formation of microparticles and the generation of procoagulant activity following DDAVP stimulation has been described [32]. It has been suggested that the final effect of DDAVP on platelets involves the facilitation of glycoprotein (Gp) IIbIIIa ligand interactions through the cytoskeleton focal adhesion protein VASP. This molecular mechanism modulates platelets binding to fibrinogen and vWF [33].

Among non-transfusional pharmacological agents within clinicians’ armamentarium for preventing and treating bleeding episodes in surgical patients, the utility of tranexamic acid is well established [29,30,34]. Tranexamic acid, as a synthetic derivative of the amino acid lysine, binds reversibly to plasminogen and thereby blocks the binding of plasminogen to fibrin and its activation and transformation to plasmin on the fibrin surface, and also inhibits circulating plasmin. Additionally, the drug enters the extravascular space and accumulates in tissues where it inhibits tissue fibrinolysis with consequent clot stabilization. Thus, this antifibrinolytic drug would be expected to prevent excessive or secondary bleeding episodes in patients with thrombocytopenia or platelet dysfunction by supporting fragile hemostatic plugs [29,34]. It is an effective method even when bleeding is not associated with laboratory signs of excessive fibrinolysis, as it was in this case [29].

In situations of high clinical priority such as possible bleeding complications during surgery caused by hemostatic derangement, a thromboelastometric assessment of hemostasis and systemic hemostatic drugs administration could be considered as a useful pre/perioperative approach as in our case [30,35]. In our patient, despite severe thrombocytopenia and an expected low increment in the platelet count after PC transfusion, optimal surgical hemostasis was successfully achieved.

Generally, the benefits of splenectomy in patients with VL are not well defined. Removing the large amounts of parasites is one of the potential benefits as, in the presence of severe splenomegaly, the levels of drugs in the spleen for the treatment of VL may not be sufficiently high to eradicate the parasite and cure the disease [36,37]. Some authors also suggest a splenectomy in patients with recurrent VL [37,38]. We believe that a splenectomy may be considered with a curative purpose for patients who develop hypersplenism and coagulation abnormalities with clinical repercussions.

## 4. Conclusions

Out of endemic areas, the recognition and management of visceral leishmaniasis can be challenging. In the era of mass tourism, war conflicts, and population movements, symptoms such as fever, hepatosplenomegaly, anemia, or pancytopenia should arouse the clinician’s suspicion of visceral leishmaniasis. A real-time bedside hemostasis assessment and a global assay together with systemic hemostatic drug treatment appears to be a useful approach in patients with coagulation abnormalities undergoing surgery. A splenectomy as a surgical treatment of hypersplenism of unclear etiology and the prevention of possible organ rupture in our case proved to be therapeutic and at the same time also a diagnostic procedure.

## Figures and Tables

**Table 1:** Hemostasis tests results before surgery.

Laboratory Test	Results Before Operation	Normal Value
APTT	44.9	25–42 s
PT	23.1	10.4–13 s
INR	1.97	0.8–1.2
Platelet count	11	158–425 × 10^9^ /L
Hemoglobin	94.7	119–175 g/L
Fibrinogen	3.8	1.8–3.5 g/L
FII	66	70–120%
FV	48	70–140%
FVII	21	70–120%
FVIII:C	94	70–150%
FIX	87	70–120%
FX	55	70–120%
FXI	52	70–120%
FXIII	34	70–140%

**Table 2:** ROTEM results before surgery.

ROTEM Parameters	Before Operation	Reference Value
EXTEM CT	75	38–79 s
EXTEM CFT	412	34–159 s
EXTEM AA	47	63–83°
EXTEM MCF	35	50–72 mm
INTEM CT	175	100–240 s
INTEM CFT	405	30–110 s
INTEM AA	52	70–83°
INTEM MCF	33	50–72 mm
FIBTEM MCF	16	9–25 mm

CT: clotting time; CFT: clot formation time; AA: alpha angle; MCF: maximum clot firmness.

## Data Availability

All the data are available from the corresponding author upon reasonable request.
